# Soft Perfusable Device to Culture Skeletal Muscle 3D Constructs in Air

**DOI:** 10.1021/acsabm.3c00215

**Published:** 2023-06-21

**Authors:** Federica Iberite, Marco Piazzoni, Daniele Guarnera, Francesco Iacoponi, Silvia Locarno, Lorenzo Vannozzi, Giacomo Bolchi, Federica Boselli, Irini Gerges, Cristina Lenardi, Leonardo Ricotti

**Affiliations:** † The BioRobotics Institute, Scuola Superiore Sant’Anna, Piazza Martiri della Libertà 33, 56127 Pisa, Italy; ‡ Department of Excellence in Robotics & AI, Scuola Superiore Sant’Anna, Piazza Martiri della Libertà 33, 56127 Pisa, Italy; § Department of Physics, Università degli Studi di Milano, Via Celoria 16, 20133 Milano, Italy; ∥ Department of Biomedical, Surgical and Dental Sciences, Università degli Studi di Milano, 20100 Milano, Italy; ⊥ Tensive s.r.l, Via Timavo 34, 20124 Milan, Italy

**Keywords:** 3D culture, skeletal muscle tissue engineering, perfusion, porous scaffold, soft biomaterial

## Abstract

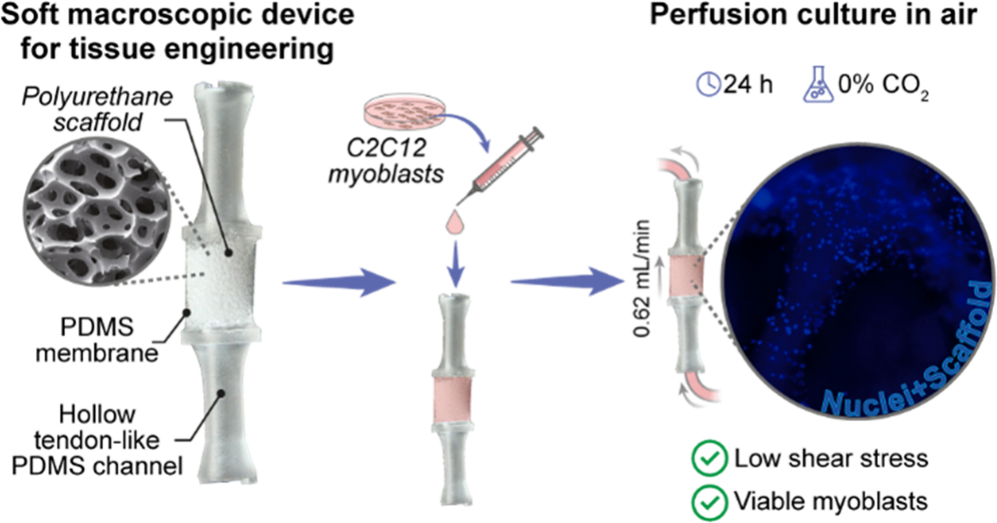

Devices for *in vitro* culture of three-dimensional (3D) skeletal muscle tissues have multiple applications, including tissue engineering and muscle-powered biorobotics. In both cases, it is crucial to recreate a biomimetic environment by using tailored scaffolds at multiple length scales and to administer prodifferentiative biophysical stimuli (*e.g.*, mechanical loading). On the contrary, there is an increasing need to develop flexible biohybrid robotic devices capable of maintaining their functionality beyond laboratory settings. In this study, we describe a stretchable and perfusable device to sustain cell culture and maintenance in a 3D scaffold. The device mimics the structure of a muscle connected to two tendons: Tendon–Muscle–Tendon (TMT). The TMT device is composed of a soft (*E* ∼ 6 kPa) porous (pore diameter: ∼650 μm) polyurethane scaffold, encased within a compliant silicone membrane to prevent medium evaporation. Two tendon-like hollow channels interface the scaffold with a fluidic circuit and a stretching device. We report an optimized protocol to sustain C2C12 adhesion by coating the scaffold with polydopamine and fibronectin. Then, we show the procedure for the soft scaffold inclusion in the TMT device, demonstrating the device’s ability to bear multiple cycles of elongations, simulating a protocol for cell mechanical stimulation. By using computational fluid dynamic simulations, we show that a flow rate of 0.62 mL/min ensures a wall shear stress value safe for cells (<2 Pa) and 50% of scaffold coverage by an optimal fluid velocity. Finally, we demonstrate the effectiveness of the TMT device to sustain cell viability under perfusion for 24 h outside of the CO_2_ incubator. We believe that the proposed TMT device can be considered an interesting platform to combine several biophysical stimuli, aimed at boosting skeletal muscle tissue differentiation *in vitro*, opening chances for the development of muscle-powered biohybrid soft robots with long-term operability in real-world environments.

## 1 Introduction

The growth of mammalian cells *in vitro* using traditional cell culture methods is still far from accurately reproducing physiological conditions found in native biological tissues.^1^ Indeed, when cultured in standard polystyrene flasks and Petri dish, cells assume an unnatural flat conformation, establish limited cell–cell interactions, and are entirely deprived of tissue micro- and macro-architecture.^2−4^ The absence of an extracellular matrix (ECM) makes it impossible to confer cells with a three-dimensional (3D) support bearing all of the mechanical, topographical, and biochemical cues necessary to trigger fundamental signaling pathways.^5^ Given this complex interplay between a cell and its surrounding, it is not surprising that technologies to culture cells in 3D structures (*i.e.*, scaffolds) constitute a hot topic, aiming at recapitulating *in vitro* specific tissue conditions typically found *in vivo*.^6−8^

Concerning 3D skeletal muscle cell cultures, several biomaterials have been tailored as scaffolds in order to mimic ECM properties, trying to recreate a suitable environment for muscle cell attachment, growth, and differentiation.^9^ Biomaterials can have different origins: natural (*e.g.*, collagen, fibrin), synthetic (*e.g.*, poly(glycolic acid), poly(lactic-*co*-glycolic acid)), or a combination of both.^10^ Synthetic polymers can also be engineered to finely adjust a multitude of biophysical parameters that foster tissue formation, such as scaffold stiffness.^11−14^ Moreover, proteins of the skeletal muscle tissue basement membrane (*e.g.*, collagen IV, laminin, and fibronectin) have been used to coat synthetic polymers, providing biochemical cues to maximize cell adhesion.^15−18^

Biophysical stimuli (*e.g.*, electrical and mechanical) that muscle cells naturally receive *in vivo* are also necessary to achieve a functional contractile construct.^19^ Among them, mechanical tension is particularly required during muscle development and maintenance since prolonged immobility leads to muscle degeneration (as occurs upon certain illnesses or in the absence of gravity).^20,21^ Hence, many protocols and platforms have been recently proposed to deliver cells with static and/or cyclic stretching, leading to aligned, well-differentiated, and hypertrophic muscle fibers.^22−24^

Despite all of these advances, scalability still represents a big challenge and a bottleneck that hampers the translation of 3D muscle cell cultures into practical fields such as skeletal muscle tissue engineering (SMTE) and biohybrid soft robotics.^15,25^ Making 3D tissues with macroscopic dimensions (cm range) remains a difficult task to achieve from the standpoint of oxygen and nutrient supply.^26,27^ This becomes critically limited in avascular constructs thicker than 150 μm, with a cellular density resembling the one found *in vivo* (∼10^9^ cells/cm^3^).^28,29^ Currently, the best solution to improve mass transport into scaffolds is the use of perfusion devices.^30,31^ Perfusion devices specifically developed for skeletal muscle cells usually consist of a porous scaffold seeded with myoblasts, which is sealed in a perfusion chamber. The latter is then connected, through an inlet and an outlet, to a fluidic circuit controlled by a peristaltic pump.^32^ Additionally, recent works presented modular systems that allow the parallelization and automatization of multiple experiments.^33,34^ In this framework, mathematical modeling techniques are often used to predict fluid flow velocity, wall shear stress (WSS), and spatial distribution of nutrients and oxygen inside the scaffold.^35,36^

Nevertheless, the conventional rigidity of perfusion chambers and the absence of an adequate scaffold clamping mechanism impede effective mechanical stimulation of the scaffold within these device configurations.^32−36^ Another critical aspect of 3D cultures is their confinement in CO_2_ incubators to keep cells alive. This feature is a considerable limitation in the field of muscle-powered robotics since it hampers biohybrid machines from operating in real-world scenarios.^25,37−40^ To date, only a handful of soft biohybrid robots performing in air were reported in the literature.^41,42^ Morimoto et al. presented a novel system wherein a contractile engineered muscle construct was encapsulated within a hydrogel shell composed of collagen. Although the robot was able to actuate in air, it succeeded in performing continuous actuation for just 1 h before drying out. The use of hydrogels as muscle cell protective caps cannot prevent liquid evaporation, thus compromising long-term applicability in air.

In this paper, we propose an approach that tackles some of the key bottlenecks in the state of the art of biohybrid systems. The proposed device has a structure resembling the anatomy of a skeletal muscle between two tendons (Tendon–Muscle–Tendon, from now on called TMT). This was achieved by integrating a soft tubular elastomeric porous polyurethane (PU) scaffold within a flexible elastomeric device. The scaffold is encased within a compliant silicone membrane, and it was connected at its two ends to two tendon-like hollow channels. These channels enable interfacing with a fluidic circuit for medium supply, or with a stretching device for mechanical stimulation of myoblasts. We aimed to characterize and evaluate the performance of the proposed TMT device and to demonstrate the feasibility of maintaining myoblast cultures for a period of 24 h outside of a CO_2_ incubator. Keeping skeletal muscle constructs in air opens new avenues for exploiting skeletal muscle tissue-engineered constructs in real-world contexts.

## 2 Experimental Section

### 2.1 Polyurethane Scaffold

#### 2.1.1 Synthesis and Fabrication

The synthesis of the polyurethane-based scaffold was carried out as previously described in Guarnera et al.^43^ The scaffolds were kindly supplied by Tensive s.r.l. (Italy). Briefly, a polyol mixture, composed of PEG, glycerol, and Milli-Q distilled water, was mechanically mixed with the polyisocyanate Tolonate X FLO 100 (NCO index = 100) and the metallorganic catalyst dibutyltin dilaurate (DBTL) for 30 s prior to pouring the mixture in a 2 L rectangular container, where the foam was let to expand until the crosslinking point was reached (after 40–45 s). The solidified foam was placed in an oven at 40 °C for 24 h to complete the curing process. Cylindrical samples (height = 3 cm, diameter = 1 cm) were cut from the raw foam and purified according to the procedure previously described in Gerges et al.^44^ The scaffolds were stored at 4 °C until use. The amount and temperature of each reactant of the blend at the moment of mechanical mixing are reported in Table 1.

**Table 1:** Synthesis of the Polyurethane-Based Scaffold^a^^,^^b^

reactant name	weight (g), pphp	purity	temperature (°C)	supplier
PEG	16.00, 79.46	99%	80	Sigma-Aldrich
glycerol	1.72, 8.57	≥99% (GC)	80	Sigma-Aldrich
Milli-Q water	2.40, 11.96	≥99%	80	na
Tolonate X FLO 100	63.87, (NCO index = 100)	≥99%	25	Vencorex
DBTL	0.38, (0.5% w/w)	≥96.0%	25	Sigma-Aldrich

a The Amount of Each Blend Reactant and the Temperature at the Moment of Mechanical Mixing Are Reported.

b na = not available; pphp = parts per hundred parts of polyol.

#### 2.1.2 Morphological and Architectural Analyses

Scanning electron microscopy (SEM) was performed on PU scaffolds provided with a metallic coating using gold sputtering for 60 s and a current of 20 mA (Q150R ES, Quorum Technologies). SEM scans were performed by setting a beam voltage of 10 kV at low vacuum (60 Pa; Phenom XL, Nanoscience Instruments, Waltham).

Microtomography (μCT) 3D scans were obtained through a customized cone beam system (Tomolab; cone beam energy = 40 kV, power = 200 μA, exposition time = 1.5 s). The size of the tomographic projections was 2004 × 1335 pixels, with a final resolution of 8 μm. The software Cobra Exxim was used for slice reconstruction and correction, while the image binary processing was performed as described by Otsu et al.^45^ The images were generated and analyzed through the plug-in BoneJ within the software Amira (Thermo Fisher Scientific, Waltham).^46,47^ The file that underwent such analysis had a volume of 1000 pixels per side (8 mm).

#### 2.1.3 Mechanical Characterization

Uniaxial compression tests for Young’s modulus calculation were performed using an Instron Series 4460 mechanical loading structure equipped with a ± 10 N load cell. Compression velocity was set equal to 2 mm/min. PU scaffolds (height = 1 cm) were tested in a dry (dry PU, *N* = 4) and wet state (wet PU, *N* = 4). Wet PU scaffolds were immersed in phosphate-buffered saline without Ca^2+^ and Mg^2+^ (PBS, Corning, 21-040-CMR) at 37 °C for 24 h and kept immersed in PBS during the test. The specimen diameter was measured with a caliper to evaluate the swelling ratio. Stress was evaluated as the ratio of the force measured by the load cell to the undeformed sample cross section. Strain was determined as the ratio of the crosshead displacement to the initial sample height. Young’s moduli were obtained considering the initial elastic region of the stress–strain curve (strain up to 10%).

#### 2.1.4 Degradation Tests

PU scaffolds (height = 5 mm) for biodegradation studies were kept immersed in growth medium (GM) composed of Dulbecco’s modified Eagle’s medium (DMEM, Corning, 10-013-CV) supplemented with 10% fetal bovine serum (FBS, Sigma-Aldrich, F4135), 1% penicillin/streptomycin (P/S, Sigma-Aldrich, P0781), and 2.5 μg/mL amphotericin B (Euroclone, ECM0009D) in a CO_2_ incubator (37 °C, 5% CO_2_). The medium was changed every 48 h. Time points for analyses were set at 14, 30, and 60 days.

For weight loss calculation (*W*), a sample set (*N* = 4) was used for each time point. Before testing, the samples were rinsed in distilled water and dried at 37 °C until weight loss stabilization (∼9 h). Each sample was weighted in the dry state at day 0 (*W*_0_) and at a specific time point (*W_t_*). Weight variation was calculated as follows
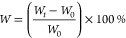
2.1For Young’s modulus calculations at compression, a sample set (*N* = 3) was used at each time point. Samples were rinsed in distilled water before testing and they were kept immersed in PBS during the test. The Young’s modulus of each sample was calculated on day 0 (*E*_0_) and at a specific time point (*E_t_*). Young’s modulus variation was calculated as follows
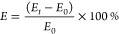
2.2

### 2.2 Cell Adhesion Tests on the Polyurethane Scaffold

#### 2.2.1 Scaffold Coating

PU scaffolds (*N* = 3, height = 2 mm) were immersed in 70% ethanol (EtOH) for 30 min, then abundantly rinsed with PBS, and treated with ultraviolet (UV) light for 30 min, to sterilize them. The UV light treatment was performed under a sterile biological hood by placing the samples in front of a UV-C lamp (Sankyo Denki, G15T8). Three conditions were tested: (1) PU scaffolds without functionalization (bare PU); (2) PU scaffolds functionalized with polydopamine (PDA); (3) PU scaffolds functionalized with PDA and fibronectin (PDA+FN).

A solution of ultrapure Tris-HCl, pH 8.0, 10 mM (Gibco, 15568-025) with 2 mg/mL dopamine hydrochloride (DA, Sigma-Aldrich, H8502) was prepared in sterile conditions. The solution was magnetically stirred in a sealed vial at room temperature (RT) until complete dissolution. Then, the scaffolds (PDA and PDA+FN) were covered with 450 μL of DA solution and incubated in an orbital shaker at 37 °C for 24 h. Scaffolds were then washed with sterile Milli-Q water 5 times to remove the PDA excess (2 min incubation at 37 °C on the orbital shaker after every wash). The excess was aspirated, and the scaffolds (PDA+FN) were coated with 200 μL of FN (Sigma-Aldrich, F4759) diluted in ultrapure Tris-HCl 10 mM pH 8 (24 h at 37 °C). The amount of FN for each scaffold was calculated to have approximately 2 μg/cm^2^, based on the known scaffold surface resulting from μCT analyses. The excess FN solution was then removed, and the samples were air-dried within a biological hood for 45 min.

#### 2.2.2 Static Cell Cultures

The cytocompatibility of the PU scaffold was previously demonstrated. For all *in vitro* experiments of this work, C2C12 murine skeletal myoblasts (ATCC, CRL-1772) were used; cells were subcultured in GM. For myoblast adhesion tests, the three conditions were compared: cell seeded on the bare PU, PDA, or PDA+FN. Cells were seeded dropwise with 200 μL of myoblast suspension (60,000 myoblasts/scaffold) in GM and incubated in a CO_2_ incubator at 37 °C for 3 h. Afterward, 1.5 mL of GM was added to each scaffold. GM was changed after 48 h, and the cultures were maintained for 3 days.

#### 2.2.3 Cell Staining and Imaging

PU scaffolds were rinsed with PBS with Ca^2+^ and Mg^2+^ (PBS+) and fixed with 4% paraformaldehyde (PFA, Thermo Scientific, 28908) in PBS+ for 20 min at RT. Afterward, samples were rinsed twice with PBS+ and permeabilized with 0.1% Triton X-100 (Sigma-Aldrich, X100) in PBS+ for 10 min at RT. The samples were then incubated for 30 min at RT in the dark with a staining solution composed of phalloidin–tetramethylrhodamine (TRITC) B isothiocyanate (1:1000, Sigma-Aldrich, P1951) and Hoechst 33342 (1:1000, Invitrogen, H3570) in 0.2% bovine serum albumin (BSA, PAN-Biotech GmbH, P06-139310). A final wash in PBS+ was performed before imaging the sample at the microscope. A Leica DMi8 microscope (Leica Microsystems, Wetzlar, Germany) was used for fluorescence image acquisition.

### 2.3 TMT Fabrication and Characterization

#### 2.3.1 Fabrication Procedure

**Figure 1:**
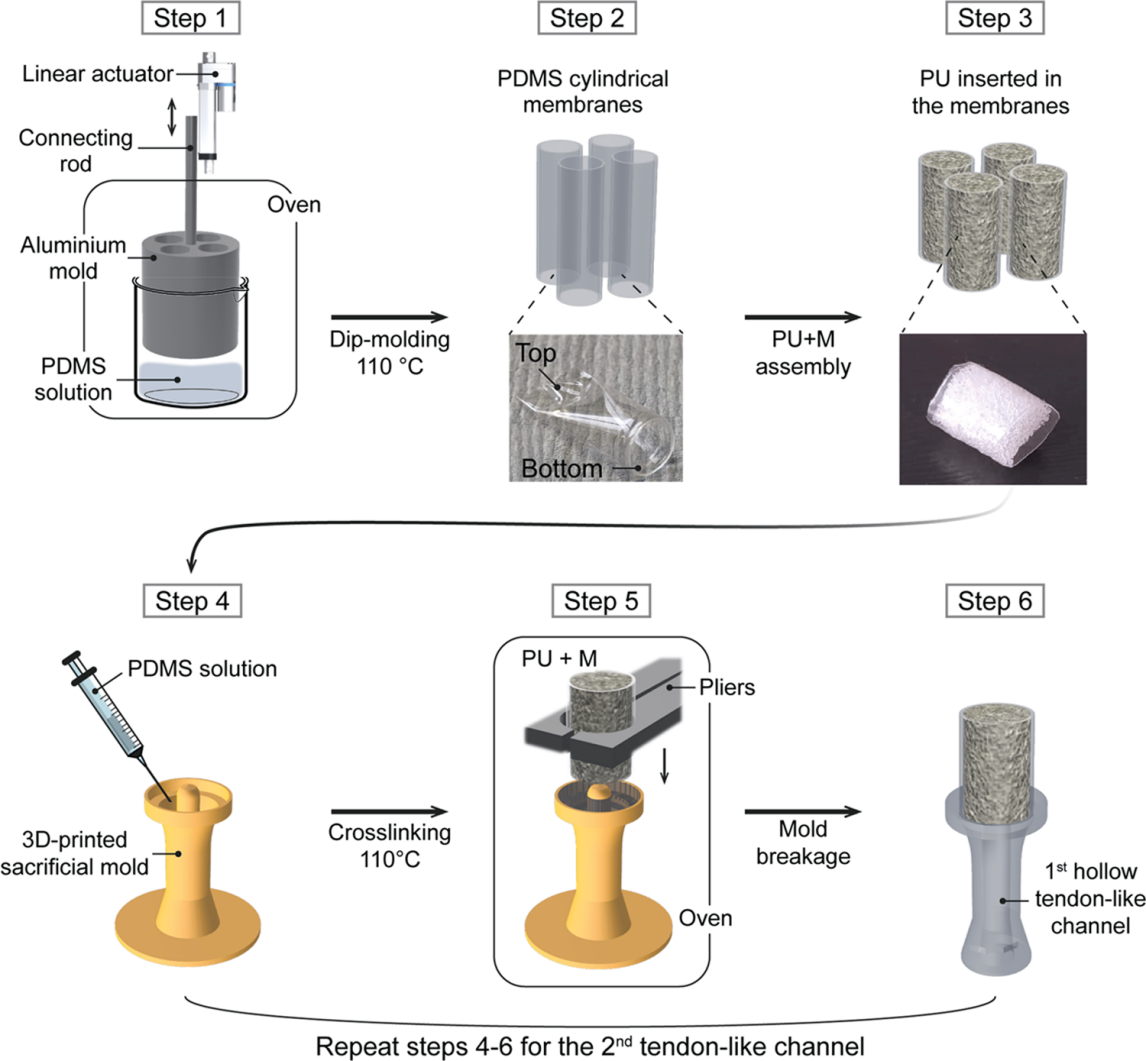
**Schematic workflow of the TMT fabrication process.** The aluminum mold is immersed in the PDMS solution (step 1); four PDMS cylindrical membranes are produced at a time and are detached from the aluminum mold (step 2); PU scaffolds are inserted in the PDMS membranes (PU+M) (step 3); a 3D-printed sacrificial mold is filled with PDMS solution (step 4); a PU+M sample is sunk on the top layer of the PDMS solution and left crosslinking in the oven (step 5); the 3D-printed sacrificial mold is shattered and the first hollow tendon-like channel is released. The procedure is repeated from step 4 to step 6 to assemble the second tendon-like channel.

The TMT device fabrication procedure is depicted in Figure 1. The polydimethylsiloxane (PDMS) membrane (M) was fabricated through a dip molding process. The dip molding setup consisted of a 3-cm-tall mold with four polished cylindrical cavities (diameter = 9 mm) connected to a linear actuator. The linear actuator was a step motor (28BYJ-48) controlled with an Arduino UNO board that moved a connecting rod up and down a rail. A PDMS solution (PDMS:crosslinker ratio of 20:1, SYLGARD 184, Dow Corning) was magnetically stirred in a beaker (20 min) and then degassed with a vacuum pump (20 min). The aluminum mold was preheated in an oven (1 h at 110 °C) and then lowered in the PDMS solution (velocity = 0.25 mm/s) (step 1). Once fully submerged, the mold remained in place for 35 s. Afterward, the mold was withdrawn (velocity = 0.125 mm/s) and left in the oven (1 h at 110 °C). Four PDMS membranes were detached from the mold by means of tweezers (step 2), and PU scaffolds (height = 1 cm) were gently inserted inside it (PU+M) (step 3). Then, every PU scaffold was melted with a soldering iron at both lateral sides, leaving space for accommodating the inner pillar of the 3D-printed sacrificial molds (not shown).

PDMS tendon-like channels (*T*) were molded in 3D-printed sacrificial molds (step 4). These molds were designed using CAD software (Fusion 360, Autodesk) and then printed using a stereolithographic 3D printer (Formlabs Form 2). The Formlabs Standard Clear V4 resin (Formlabs) was employed as printing material. A PDMS solution (PDMS:crosslinker ratio of 10:1) was magnetically stirred in a beaker (20 min) and then poured inside a syringe provided with a needle. The PDMS was injected at the bottom of the 3D-printed sacrificial mold, degassed with a vacuum pump (20 min), and partially cured in the oven (6 min at 110 °C). A PU+M was positioned with a triaxial shifter on top of the 3D-printed sacrificial mold and sunk in the PDMS solution for about 2 mm (step 5). The system was left still in the oven (15 min, 110 °C).

The 3D-printed sacrificial mold was shattered using a pair of nippers (step 6). Steps 4 to 6 were repeated to attach the second tendon-like channel on the opposite side of the PU+M. Finally, the assembled TMT device was postcured in the oven (40 min, 110 °C).

#### 2.3.2 Mechanical Properties Characterization

Uniaxial compression tests and Young’s moduli calculation on the PU scaffold encased within the PDMS membrane in a dry (*N* = 4) and a wet state (*N* = 4) were performed as described in Section 2.1.3

For dynamic tensile tests, the TMT devices (*N* = 3) were filled with DMEM at 37 °C for 24 h before testing and kept immersed in warm DMEM during the test. Tests were performed with a load cell of ± 10 N, using a frequency of 0.5 Hz^48,49^ and a strain of 15%^49,50^ of the scaffold length for 10,000 cycles. The parameters were selected to simulate the conditions of previously established protocols used to align C2C12 cells and enhance myosin accumulation.^48,49^ Integrity controls of TMT devices were performed after 0, 1,000, 5,000, and 10,000 cycles, evaluating any DMEM leakage on a white blotting paper. Softening/hardening degrees were calculated as the median value of the maximum stress in the initial 100 cycles (max σ_*i*_ median) minus that of the final 100 cycles (max σ_f_ median), as described in eq 2.3 (Shapiro–Wilk normality test; Wilcoxon matched-pairs signed rank test, *p* < 0.0001).
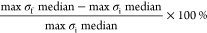
2.3

### 2.4 TMT Device Perfusion and Biological Characterization

#### 2.4.1 Computational Fluid Dynamic Simulations

Computational fluid dynamic (CFD) simulations were performed on two different representative volume elements (RVE) extracted from the PU scaffold μCT scans (see Section 2.1.2). A first RVE was used to evaluate the wall shear stress (WSS) along the scaffold (dimensions 1 × 1 × 8 mm); a second RVE was used to evaluate the percentage of scaffold volume covered by velocity values lower than 1.6 mm/s (dimensions 8 × 8 × 2 mm). The steady-state laminar flow considered for these analyses is described by the Navier–Stokes mathematical model
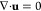
2.4
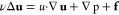
2.5where *u* expresses the velocity field, *p* is the pressure, *f* indicates the body forces, and *ν* represents the kinematic viscosity. The fluid was considered as DMEM with 5% of FBS whose properties were obtained from Poon et. al.^51^ and modeled as incompressible and Newtonian. COMSOL 6.0 Multiphysics was used to mesh the fluid domain. The tetrahedron size (10^–5^ m) and the level of the CAD degree of finish (10^4^ CAD triangular polygons) are described in Guarnera et al.,^43^ and the convergency rate was imposed equal to 10^–5^. A convergence analysis was performed for each model (Figure S1). A no-slip condition was imposed at the scaffold walls and at the RVE lateral boundaries, while the atmospheric pressure was considered a boundary condition at the outlet cross section. As the input of the simulations, an inlet flow rate spanning from 0.022 to 2.2 mL/min was imposed, mimicking the operational threshold of the peristaltic pump.

#### 2.4.2 Scaffold Perfusion and Cell Cultures

**Figure 2:**
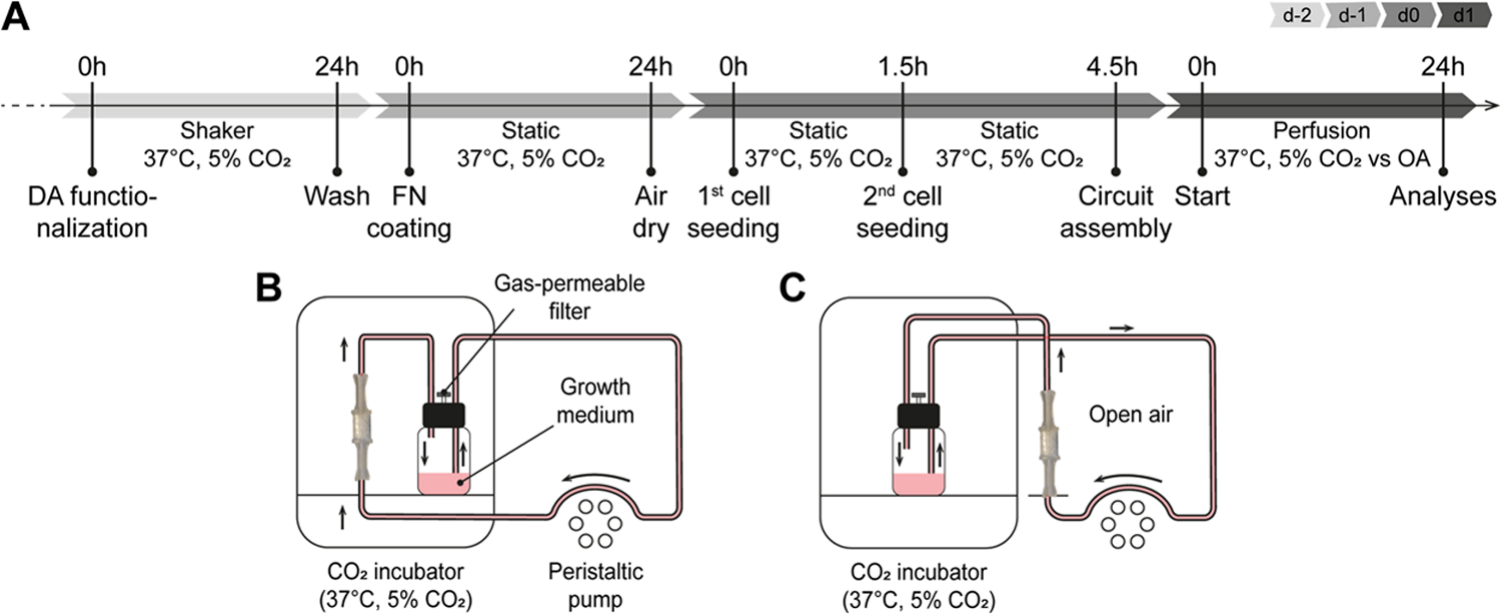
**Schematic timeline and experimental setups used for perfusion and cell culture within the TMT device.** (A) Depiction of the experimental time points, procedures, and conditions. DA: dopamine; FN: fibronectin; dX: day X; RT: room temperature; OA: open air. Schemes of the setups used for the perfusion culture of the TMT devices (B) inside (TMT in) and (C) outside (TMT out) the CO_2_ incubator.

For cell cultures under flow perfusion, an IPC-N digital multichannel peristaltic pump (Ismatec, ISM939) was used, provided with autoclavable PharMed BPT tubes (inner diameter = 1.3 mm, Ismatec, CP95809-32, flow range = 0.022–2.2 mL/min). For the *in vitro* tests, TMT devices were initially sterilized by keeping them in 70% EtOH for 3 h, flushing them 3 times with PBS supplemented with 2.5 μg/mL amphotericin B and 1% P/S, and then flushing them once with just PBS. Then, all of the liquids were aspirated from the scaffold, and 20 min of UV treatment was performed on each side of the TMT (40 min in total). Afterward, the TMT devices were used to calibrate the pump flow rate (Figure S2). The PU scaffolds inside the TMT device were coated with PDA and FN following the same procedure described in Section 2.2.1 (Figure 2A(d-1 and d-2)). All solutions were added with a syringe directly into the scaffold. The TMT devices were air-dried within a biological hood for 1 h.

C2C12 at passage 5 were detached and resuspended in GM. The TMT devices were kept horizontally during the static seeding procedure (Figure 2A(d0)), inside a Petri dish. The seeding process was divided into two sessions, and a syringe (1 mL) with a needle (23G) was used for the procedure. In the first seeding session, 5 × 10^5^ cells were seeded in 200 μL of GM; 100 μL of GM was then added to each device, and the samples were kept for 2 h in the CO_2_ incubator to allow cell attachment. For the second seeding session, each device was rotated 180° around its longitudinal axis, and 1 × 10^6^ cells were seeded following the same procedure. The samples were incubated in a CO_2_ incubator for an additional 3 h to promote cell attachment.

The devices were then ready for flow perfusion. Mini tube fittings (internal diameters = 1.6 and 2.5 mm; CARLO ERBA Reagents s.r.l., 9.207 297) were used to connect the TMT tendon-like channels to the pump tubes. The GM (7 mL for each sample) was kept in one glass reservoir for each sample, provided with 3-port connection caps (Duran, 1129751) and a pressure equalization syringe filter (0.2 μm in PTFE, Duran, 1137801) (Figure 2B,C). All of the reservoirs with the GM were kept at 37 °C for the whole experiment. The flow rate was set at 0.62 mL/min, and the test was stopped after 24 h (Figure 2A(d1)).

#### 2.4.3 Cell Staining and Viability Assessment

At d1, PU scaffolds were gently isolated from the TMT devices by separating them from the two tendon-like channels and the PDMS membrane. Each PU scaffold was divided into three sections (height ∼ 3 mm each) to facilitate the staining process and the subsequent acquisition of fluorescence images. The slices were then transferred to a 48-well plate, and the staining process was performed as described in Section 2.2.3.

Cell viability in the TMT devices was assessed by measuring lactate dehydrogenase (LDH) released in the culture medium after 24 h (d1) normalized on the total DNA. For the LDH assay, Lactate Dehydrogenase Activity Assay Kit (Sigma-Aldrich, MAK066) was used according to the manufacturer’s protocol. First, all of the medium was collected from each sample and quantified (*V*_tot_, mL). The volume taken from each *V*_tot_ to measure LDH activity (*V*_exp_, mL) was chosen so that the absorbance values were within the linear range of the standard curve. LDH release was then measured on a VICTOR X microplate reader (PerkinElmer, absorbance at 450 nm).

For DNA quantification, PU scaffolds were gently isolated from the TMT devices by separating them from the two tendon-like channels and the PDMS membrane. The scaffolds were then transferred to a 48-well plate and rinsed twice with PBS+. Cells were lysed by adding 500 μL of nuclease-free water to each sample and treating the scaffolds with three freeze–thaw cycles at −20 and 37 °C, respectively. The DNA amount in cell lysates was measured by using the Quant-iT PicoGreen dsDNA Assay Kits (Invitrogen, P11496), following the manufacturer’s instructions. The DNA amount was proportional to fluorescence intensity, which was measured on a VICTOR X microplate reader (excitation/emission of 485 nm/535 nm).

Equation 2.6 was used to measure LDH activity normalized on the amount of total DNA for each sample
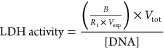
2.6where *R_t_* is the reaction time (min), *B* is the amount of NADH moles generated, and [DNA] is the DNA concentration (μg/mL).

### 2.5 Statistical Analyses

Data analyses were performed using the GraphPad Prism 8 software. Statistical tests used for each experiment are specified in the caption of the corresponding results. The significance threshold was set at 5% and computing a two-tailed p-value. Regarding box plots, boxes show the median value, 25^th^ and 75^th^ quartile ± Tukey whiskers (1.5 times the interquartile range, IQR).

## 3 Results and Discussion

### 3.1 Polyurethane Scaffold Characterization

**Figure 3:**
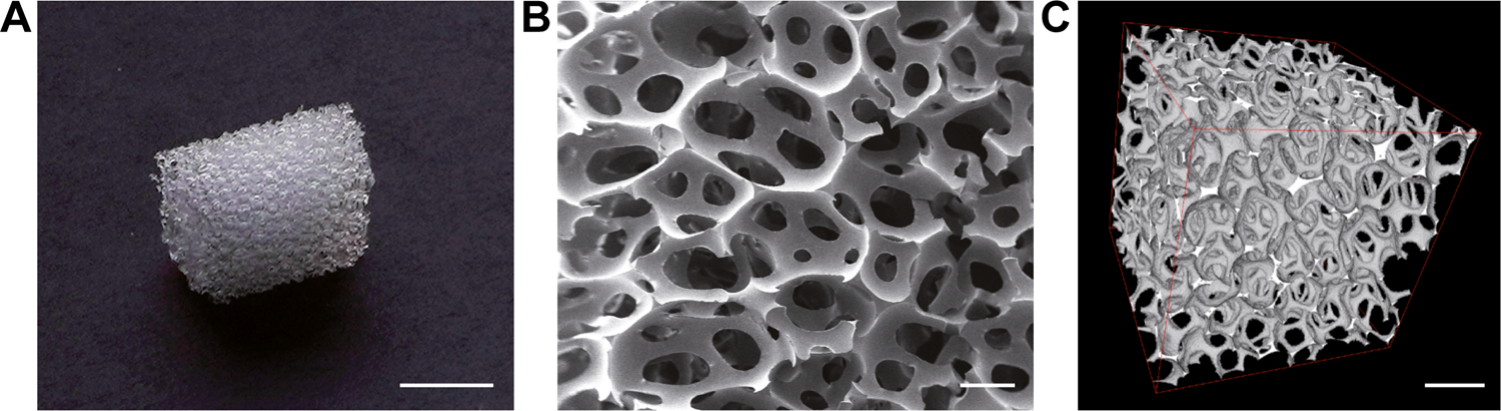
**Morphological characterization of the polyurethane scaffold.** (A) Macroscopic picture of the scaffold (scale bar = 5 mm); (B) representative SEM image of the scaffold, showing the interconnected network of pores and cavities (scale bar = 200 μm); (C) μCT 3D scan of the scaffold structure (scale bar = 200 μm).

The polyurethane (PU) scaffold (Figure 3A) constituted the core of the TMT device, hosting skeletal muscle cells. By means of different imaging techniques, it was possible to scan in detail the scaffold architecture, both qualitatively and quantitatively. In particular, from SEM images (Figure 3B), a clear distinction between cavities and pores could be observed. The cavities were large semispherical sub-structures (∼1 mm) whose surfaces were studded with smaller holes (*i.e.*, the pores). The cavity walls (*trabeculae*) looked very smooth and neat and provided a vast area that cells could exploit for adhesion on the scaffold. The pores, on the other hand, enabled fluid perfusion throughout the whole structure. μCT 3D scans (Figure 3C) provided information on the overall porosity (93.3%) and pore diameter (637 ± 188.7 μm).

Pore interconnectivity is a key requirement for nutrient diffusion and cell migration throughout the scaffold.^52^ The PU scaffold resulted in an open-cell foam (Figure 3B) with an average intrinsic permeability of 1.71 × 10^–9^ m^2^ (median value; Figure S3), guaranteeing that fluids can effectively move throughout its structure.^53^Table 2 provides a concise summary of the scaffold morphology data extracted from the μCT scans.

**Table 2:** Polyurethane Scaffold Features Extracted from the μCT Analysis

porosity [%]	trabecular thickness [μm]	pore diameter [μm]
93.3%	138 ± 64.4	665.7 ± 129

**Figure 4:**
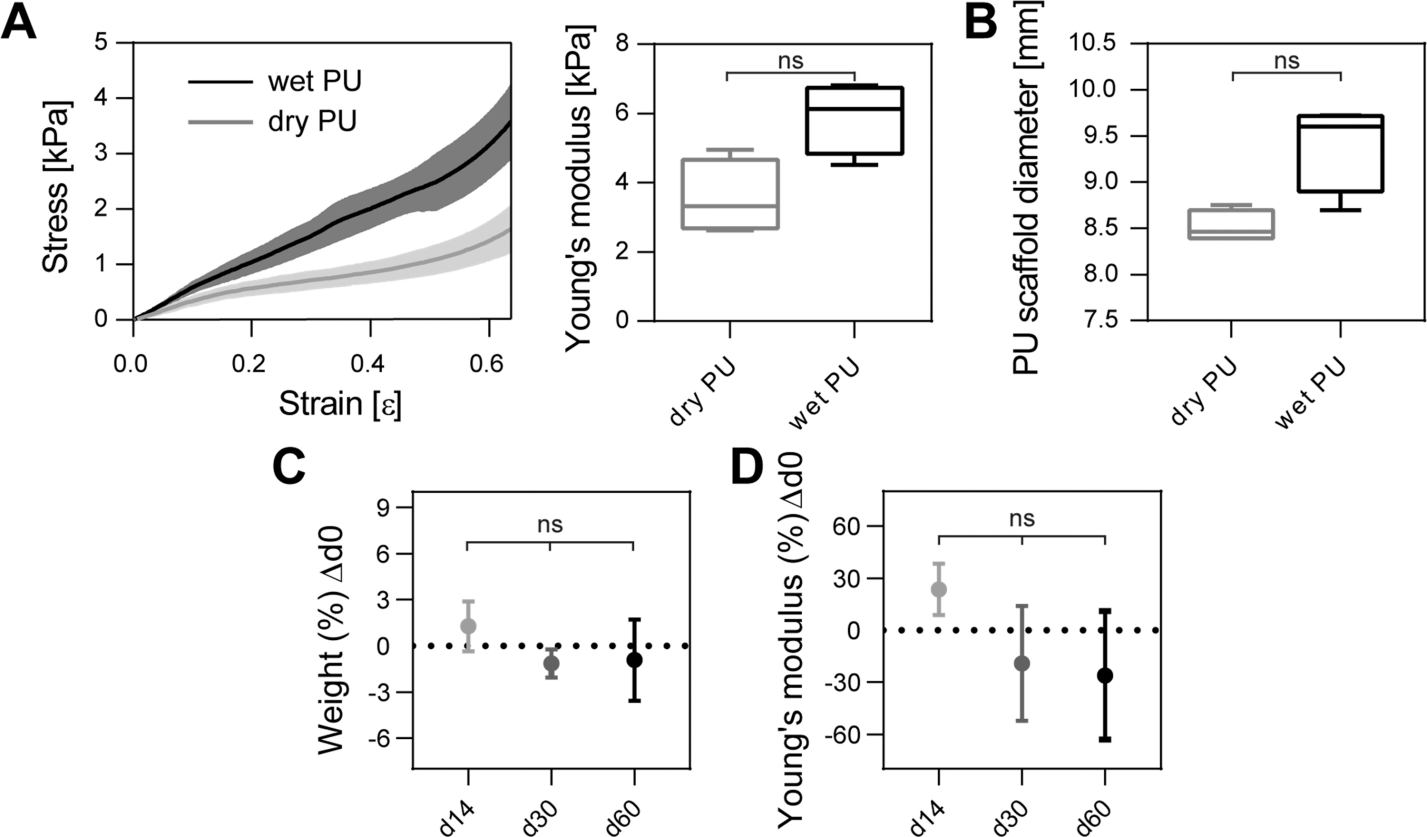
**Mechanical characterization and degradation tests of the polyurethane scaffold.** (A) Stress–strain curves of dry (gray, dry PU) and wet (black, wet PU) PU scaffolds (left; data shown as median ± SD) and Young’s modulus distributions of dry and wet PU (right) (Wilcoxon matched-pairs signed rank test). (B) Scaffold swelling ratio (Wilcoxon matched-pairs signed rank test). Results of the scaffold degradation test after 14, 30, and 60 days in DMEM at 37 °C and 5% CO_2_, reported as a trend in the variation of (C) scaffold weight and (D) scaffold Young’s modulus (Kruskal–Wallis with Dunn’s multiple comparisons test). ns = *p* > 0.05.

The stiffness of a scaffold plays a crucial role in modulating cell behavior and facilitating the differentiation of cells into skeletal muscle tissue. For this reason, uniaxial compressive tests were performed on both dry (dry PU) and wet PU scaffolds (wet PU; *i.e.*, after immersion in PBS at 37 °C for 24 h) to evaluate their compressive modulus. Stress–strain curve trends of dry and wet PU had a similar shape (Figure 4A), with an initial linear regime followed by slight plastic deformation, typical of elastomeric materials.^54^ A larger Young’s modulus value was found for the wet PU (∼5.9 kPa, median value) compared to the dry PU (∼3.5 kPa, median value), even if not statistically significant (*p* > 0.5). The similar results in terms of stiffness between the dry and wet conditions can be explained by low water absorption, as attested by the small change in scaffold diameter when immersed in PBS (Figure 4B). These values are not too far from the Young’s modulus of a natural mouse-derived muscle, which is around 12 ± 4 kPa.^12^ Furthermore, substrates Young’s modulus of around 6 kPa were found to support the self-organization of C2C12 myoblasts into aligned myotubes.^14^ The construction of biohybrid robots may benefit from the low stiffness of the scaffold, potentially resulting in reduced resistance to contraction and offering advantageous prospects.^25,55−57^

Scaffold stability over time is essential for long-term SMTE and biohybrid robotics applications. After 2 months in a culture environment (*i.e.*, immersed in DMEM at 37 °C and 5% CO_2_) PU scaffolds showed nonsignificant variation in terms of weight (≤−3%, Figure 4C), demonstrating that no relevant material degradation occurred. On the other hand, the Young’s modulus showed a slightly decreasing trend (Figure 4D), most probably due to polymer relaxation over time. However, such a decrease was not statistically significant compared to day 0. These results showed that the proposed scaffold can endure cell culture conditions over a long period (at least 2 months) with no relevant variations.

### 3.2 Evaluation of Muscle Cell Adhesion on Coated Scaffolds

**Figure 5:**
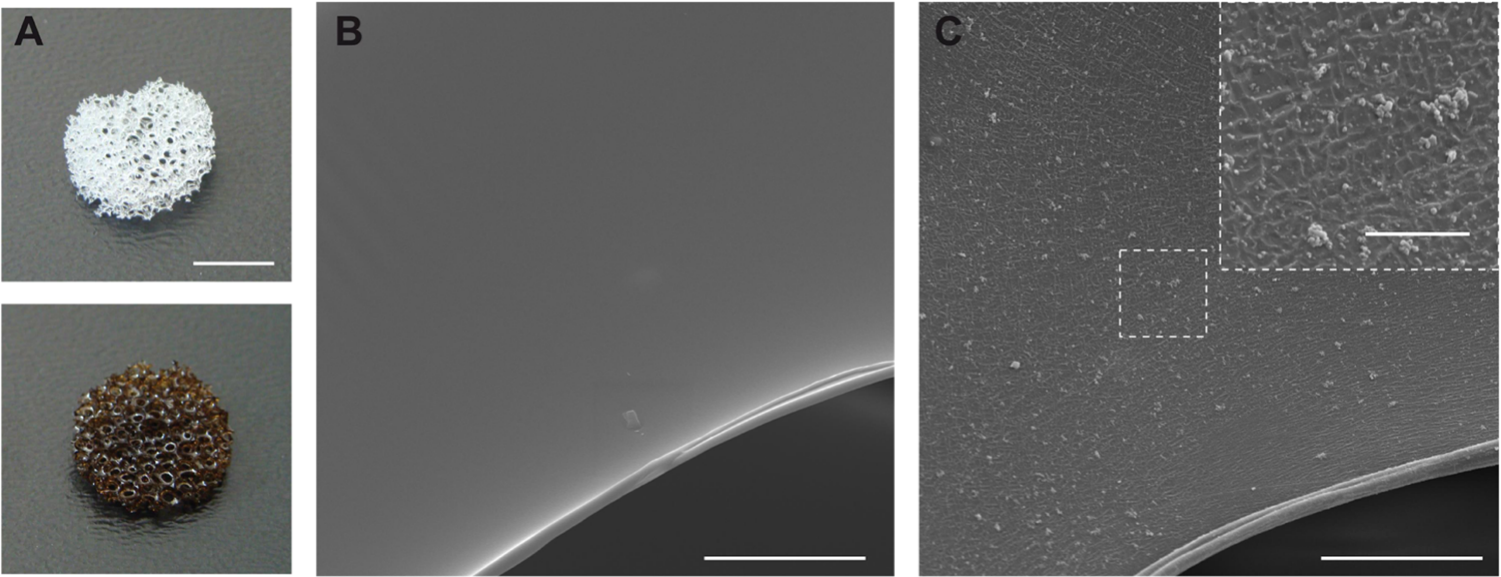
**Polyurethane scaffold coating with polydopamine.** (A) Representative pictures of a noncoated PU scaffold (top) and a coated one (bottom), with an evident color change from white to dark brown, due to the presence of polydopamine (PDA). Representative SEM images of the surface of a (B) noncoated PU scaffold, and a (C) coated one, with a zoom on PDA aggregates. Scale bars are 0.5 cm in (A), 30 μm in (B) and (C), and 5 μm in the inset (high-magnification image).

Synthetic polymers offer broad and versatile possibilities in tuning their mechanical properties. Nonetheless, they usually lack bioadhesive cues.^15^ For this reason, we developed a wet chemistry methodology to foster cell adhesion and promote the penetration of cells into the porous scaffold architecture. We adapted the polydopamine coating strategy developed by Lee et al.,^58,59^ as follows. After incubating the scaffold for 24 h at 37 °C in a solution of 2 mg/mL of dopamine hydrochloride (DA), the atmospheric oxygen led to DA polymerization in polydopamine (PDA) on the PU surface. The presence of a PDA coating was demonstrated by a color change of the material from white to brown (Figure 5A), which is a sign of catechol oxidation and subsequent DA self-polymerization.^60^ A further validation of the process was the presence of several dispersed PDA aggregates on the surface of PDA-coated scaffolds (Figure 5B,C).^61^

**Figure 6:**
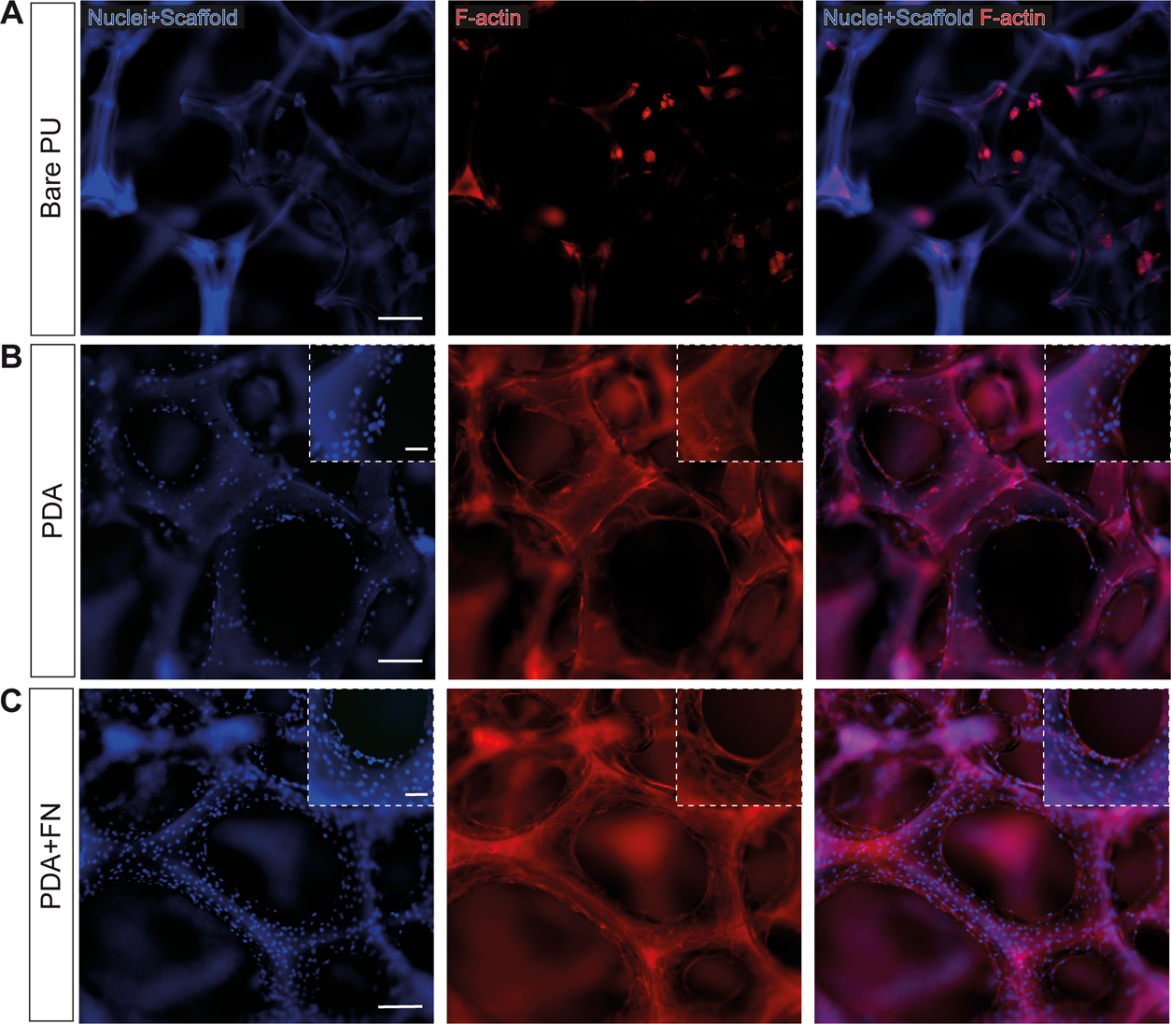
**Myoblast adhesion on coated polyurethane scaffolds after 3 days of proliferation.** (A) Representative fluorescence images of growing C2C12 myoblasts on the noncoated scaffold (bare PU), coated with (B) PDA (PDA) and (C) FN (PDA+FN). Red: F-actin (phalloidin-TRITC); blue: nuclei (Hoechst) and PU scaffold. Scale bars are 100 μm in the main images and 50 μm in the insets (high-magnification images).

The assessment of cell adhesion on the coated scaffold was performed by seeding C2C12 myoblasts. After 3 days in GM, cell spreading and adhesion were analyzed by staining for F-actin (Hoechst was used to counterstain cell nuclei) (Figure 6). Three distinct experimental conditions were compared: (1) noncoated PU scaffolds (bare PU), (2) coated with PDA (PDA), (3) coated with PDA and subsequently coated with 2 μg/cm^2^ FN (PDA+FN). In the absence of any coating, myoblasts were not able to properly adhere to the PU surface, creating scattered clusters of a few cells (Figure 6A). The presence of PDA may have played a role in reducing the hydrophobicity of the PU scaffolds and addressing the absence of adhesive sites, as evidenced by the presence of distributed and elongated cells (Figure 6B) and suggested by other recent literature studies.^61−63^ However, the highest cell adhesion was observed with the absorption of FN over the PDA layer, which was attested by the presence of a homogeneous myoblast sheet all over the scaffold surface (Figure 6C). FN is an essential glycoprotein particularly abundant in the muscle-specific ECM.^64^ It is known to enhance myoblast attachment thanks to the presence of RGD peptides (Arg-Gly-Asp) in its sequence.^65^ RGD peptides are the most frequent cell adhesion sites found in the ECM, and they have been extensively used to functionalize different materials to create cell-friendly microenvironments. The better adhesion of the myoblasts on the PDA + FN sample can be caused by the increase in cell adhesion sites, due to the presence of RGD peptides that interact directly with myoblast integrins.

All of these results proved the efficacy of the proposed surface modification strategy in creating a coating suitable for cell adhesion on the porous PU scaffold. The combination of PDA and FN was used in subsequent experiments.

### 3.3 TMT Device Design and Characterization

**Figure 7:**
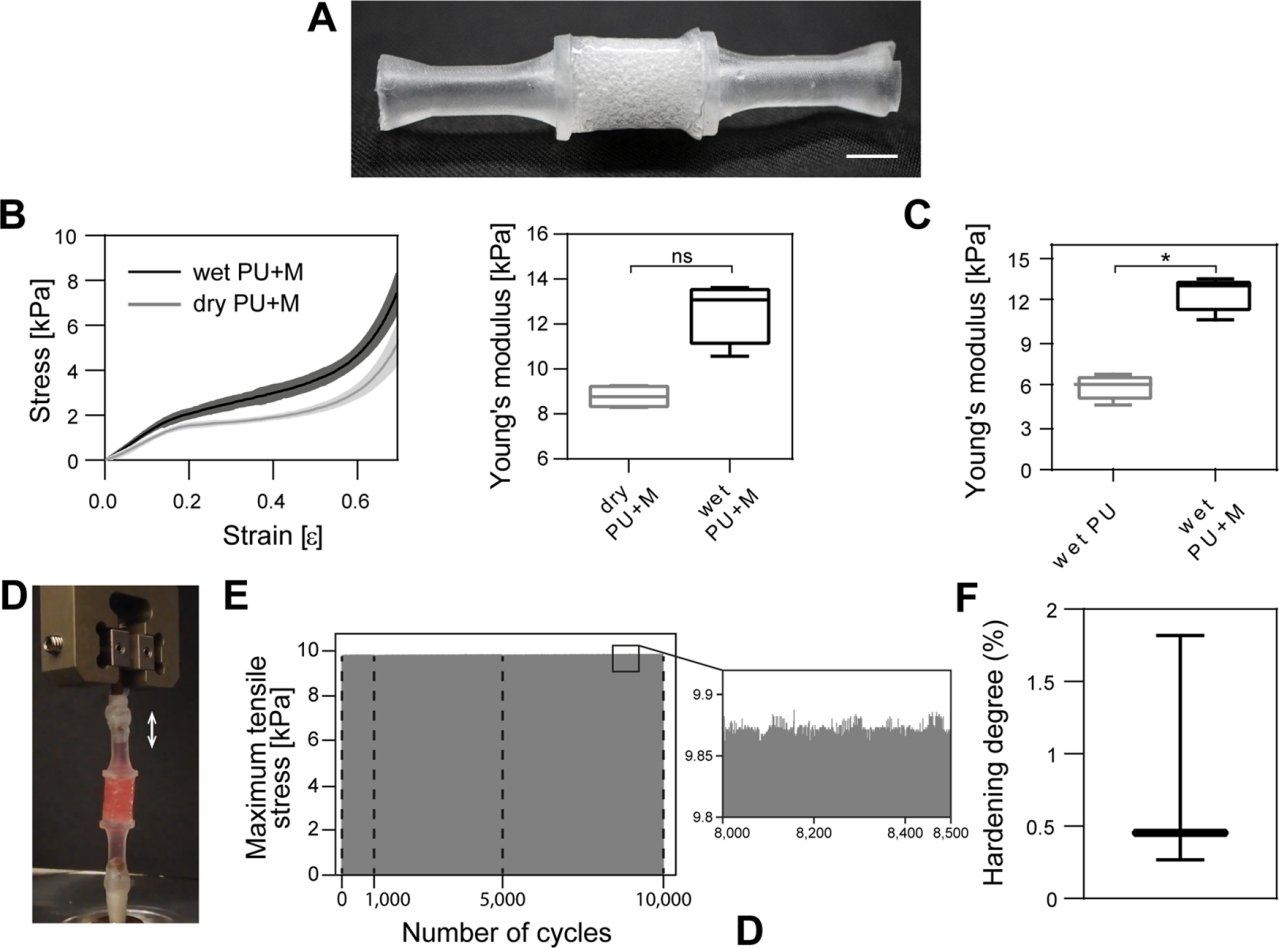
**TMT device mechanical characterization.** (A) Picture of the TMT device. Scale bar is 0.5 cm (B) Stress–strain curves of dry (gray, dry PU+M) and wet (black, wet PU+M) PU scaffold inserted in the PDMS membrane (left; data shown as median ± SD) and relative Young’s moduli distributions (right; Wilcoxon matched-pairs signed rank test, ns = *p* > 0.05). (C) Comparison between Young’s moduli distributions of the wet PU and wet PU+M (Mann–Whitney test, *p ≤ 0.05). (D) Representative plot of the cyclic uniaxial tensile tests on the TMT device with a focus showing the homogeneity of the maximum stress. Applied strain: 15%, frequency: 0.5 Hz. Black dashed lines represent the integrity checks. Picture depicting the experimental setup (E) and hardening degree distribution of the TMT device (F).

The TMT device was designed to provide a 3D culture of skeletal muscle cells in the PU scaffold with the following features: mechanical stimulation, perfusion with culture medium, and an aseptic environment to sustain cell viability in air (out of the CO_2_ incubator) (Figure 7A).

To evaluate the influence of the PDMS membrane on the overall device stiffness, compressive uniaxial tests were performed. Consistently with the data obtained on PU alone (Figure 4A), no significant variation of Young’s modulus was observed between the dry and the wet PU encased within the PDMS membrane (dry and wet PU+M; Figure 7B). Most importantly, the PDMS membranes fabricated with the dip molding procedure were compliant, thanks to a small wall thickness (100–120 μm) (Figure S4). Indeed, the PDMS membrane (wet PU+M) did not rigidly withstand the imposed deformation, only slightly increasing the *wet* PU Young’s modulus from 6 kPa (median value) to ∼13 kPa (wet PU+M, median value) (Figure 7C).

It is desirable that a device for SMTE allows mechanical stimulation of the developing skeletal muscle tissue. Tensile strain is a well-documented stimulus able to boost myoblast differentiation *in vitro* by mimicking the physical exercise of natural skeletal muscle. In this context, several stimulation protocols have been reported.^66^ More specifically, cyclic uniaxial strain (10–15% amplitude), with alternating phases of extension and relaxation, fosters myoblast differentiation by pushing myoblast alignment and overexpression of sarcomeric proteins.^48−50^ In this view, to assess the TMT device resistance to fatigue, cyclic uniaxial tensile tests were performed. Cell culture conditions were simulated by filling the TMT device with cell culture medium at 37 °C (Figure 7D). Samples were repeatedly stretched for 10,000 cycles, with a strain of 15% of the PU height, at a frequency of 0.5 Hz (Figure 7E).^48−50^ The maximum detected stress (∼9.9 kPa) remained stable over 10,000 cycles, showing a slight hardening degree of 0.45% (median value; Figure 7D,F). Additionally, no medium leaks were detected at any integrity check. These results confirmed that the PDMS tendon-like channels managed to crosslink around a PU+M creating a sealed interface among the three components (PU+M+T). The TMT device can therefore undergo a potential mechanical stimulation protocol in conditions compatible with cell culture maintenance.

### 3.4 Open-Air Application of the TMT Device

To demonstrate the ability of the TMT device to maintain cells alive in air, a set of devices was seeded with myoblasts and kept outside the CO_2_ incubator. Unidirectional medium perfusion was provided with a peristaltic pump. Cell adhesion and viability were assessed after 24 h and compared with those found in TMT devices kept in a standard culture environment (*i.e.*, inside the CO_2_ incubator). The results of this experiment are reported in the following subsections.

#### 3.4.1 Computational Fluid Dynamic Analyses to Estimate the Optimal Medium Flow Rate

Computational fluid dynamics (CFD) analyses were employed to determine the optimal flow rate, guided by the peristaltic pump, in order to prevent excessive shear stress while ensuring extensive nutrient coverage across the scaffold. This was achieved by establishing suitable fluid velocity values. The simulation technique has already been used and validated by Guarnera et al.,^43^ with the same scaffold. In this work, the estimated permeability obtained through different RVEs was compared to experimental data (Figure S1).

**Figure 8:**
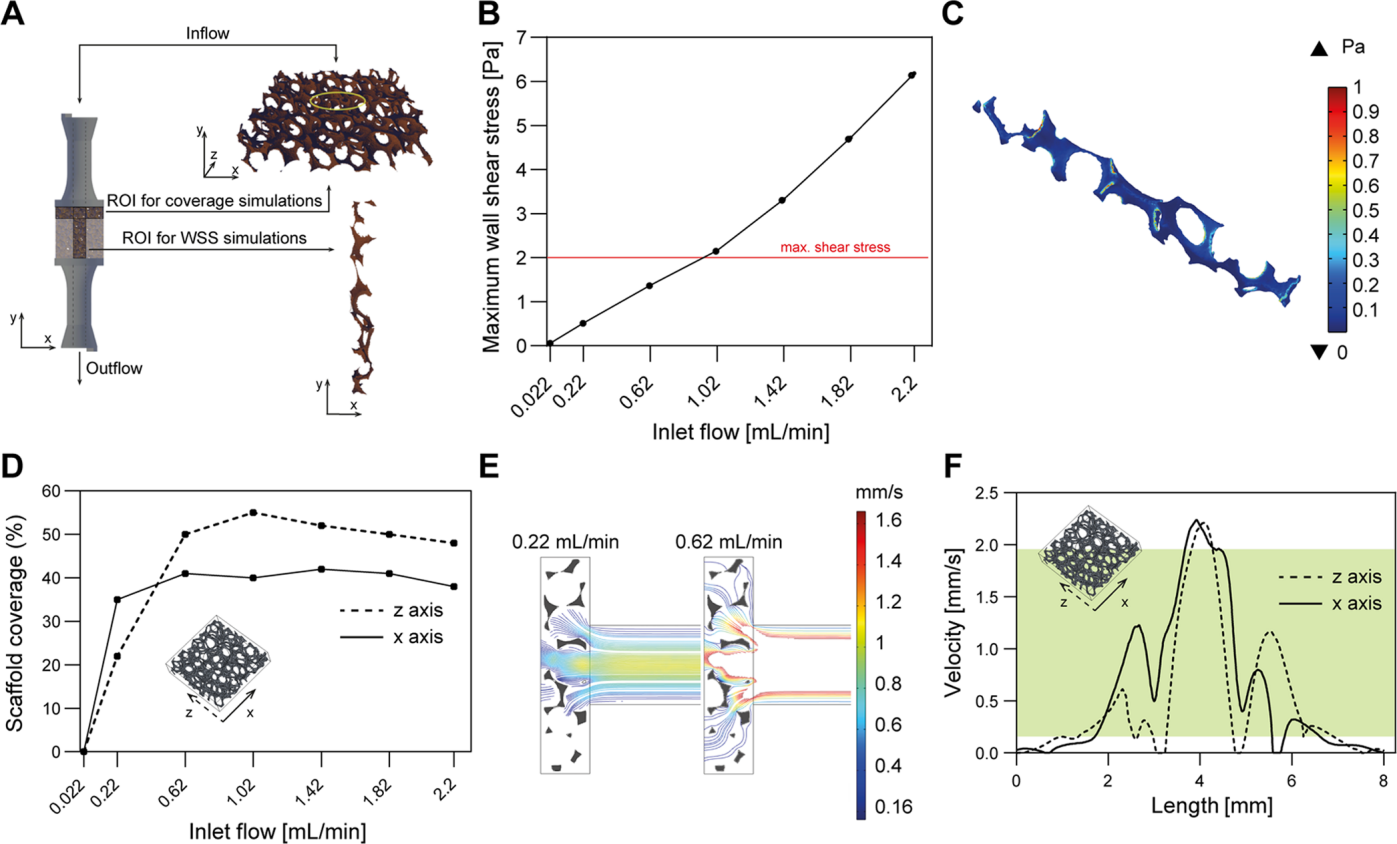
**Computational analyses.** (A) TMT CAD representation and RVEs used for simulations. (B) WSS_max_*vs* flow rate at the inlet. The maximum admissible WSS threshold (over which cells risk to detach) is highlighted with a red line. (C) 3D contour plot of the estimated WSS over the PU scaffold walls, for an inlet flow rate equal to 0.62 mL/min. (D) Areas of PU scaffold covered by optimal values of velocity at increasing flow rates. (E) Comparison between two different inlet flow rates in terms of optimal velocity (0.1–2 mm/s) streamlines, over the PU scaffold transversal RVE. (F) Distribution of velocity along two orthogonal pathways (*x*-axis, bold line; *z*-axis, dashed line) within the transversal RVE imposing a flow rate equal to 0.62 mL/min. The green band represents the optimal velocity range (0.1–2 mm/s).

A longitudinal RVE (Figure 8A, bottom) was used to estimate WSS values along the scaffold surface at increasing flow rates imposed at the TMT device inlet. In this case, the selected RVE runs along the whole length of the scaffold and it is extracted from its center (where the flow is stronger) to take into account the worst-case scenario. Simulation results in terms of maximum wall shear stress (WSS_max_) are shown in Figure 8B. A maximum threshold of 2 Pa was set as the value above which cell detachment has been observed in previous reports.^67−69^ Although just in a few points of the RVE, GM flow rates greater than 0.62 mL/min exceeded the WSS threshold. Figure 8C shows the WSS 3D contour plot when a flow rate equal to 0.62 mL/min was imposed at the inlet.

A transversal RVE (Figure 8A, top) allowed us to investigate the velocity field inside the PU scaffold at increasing GM flow rates (from 0.022 up to 2.2 mL/min). The velocity module was computed in two orthogonal reference lines, one along the *x*-axis direction and the other one along the *z*-axis direction. Results are expressed in terms of the percentage of scaffold areas hit by the fluid with specific velocity values (0.1–2 mm/s) (Figure 8D). These values have been shown as the optimal to guarantee proper nutrient exchange, since they are similar to those measured in the cardio-circulatory system.^70−74^ The coverage percentage increased by increasing the flow rate, up to a maximum of ∼55% on the *z* axis and ∼40% on the *x*-axis. In Figure 8E, the fluid velocities are shown in the scaffold cross section for two different values of inlet flow rate, namely, 0.22 and 0.62 mL/min. In the first case, in many parts of the scaffold section, flow streamlines did not reach the minimum value of the optimal velocity range (*i.e.*, 0.1 mm/s), especially at the edges of the RVE. Instead, when a flow rate of 0.62 mL/min was applied, the fluid was much more evenly distributed across the scaffold. Finally, it is worth noting that, at 0.62 mL/min, the flow was mostly concentrated in the center of the scaffold, as shown in Figure 8F where velocities values along the *x*-axis (bold line) and the *z*-axis (dashed line) are plotted. By imposing such a flow rate, the vast majority of the scaffold was invested by fluid streamlines having velocity values that fall into the above-mentioned optimal range, represented in Figure 8F by the green area.

Overall, the flow rate of 0.62 mL/min resulted to be the best compromise for the subsequent perfusion cell culture experiments. Indeed, this flow rate ensured low WSS values (<2 Pa) and a good percentage of scaffold coverage (∼50%) with optimal fluid velocity values.

The shear stress increased almost linearly with the inlet flow rate. At 0.62 mL/min, the WSS_max_ was equal to 1.39 Pa. Though below the threshold, this value is higher than other ones found in the literature for porous materials,^75,76^ probably due to the high PU scaffold porosity (93%) and to the pore diameters. However, the average value of 0.072 Pa should ensure a safe flow.^77,78^ To the best of the authors’ knowledge, the analysis of scaffold areas hit by flow with optimal velocities has never been explored. This analysis should be considered a novel methodological workflow that may be adopted in future studies focusing on perfusable scaffolds.

#### 3.4.2 Perfusion of Skeletal Muscle Cell Cultures in Air

Before seeding cells on the PU, the sterilized TMT devices were used to calibrate a proper flow rate of the peristaltic pump by perfusing sterile PBS. The significance of calibration with the devices was confirmed by the achievement of improved precision and accuracy of the pump’s performance (Figure S2).

**Figure 9:**
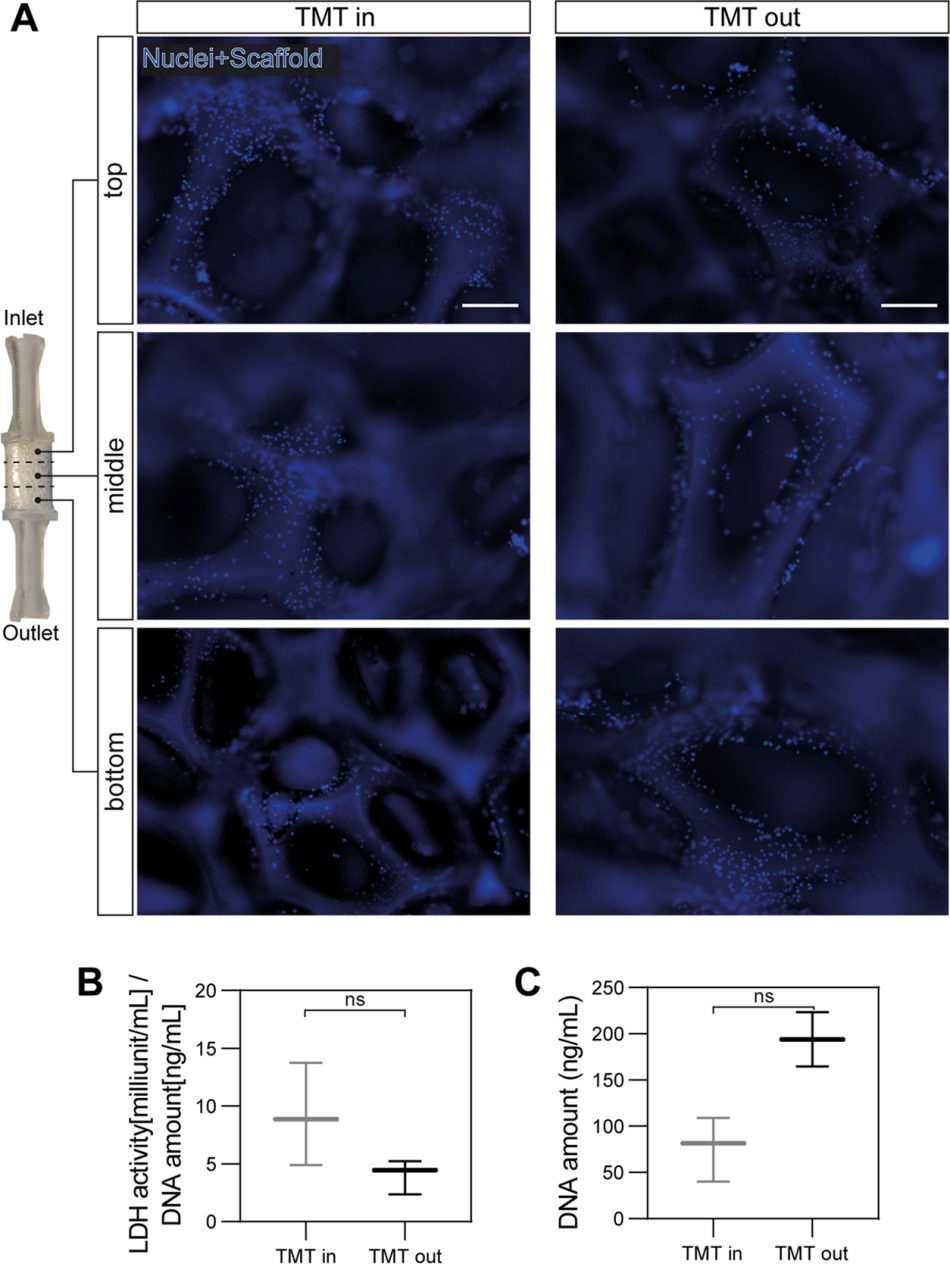
**Myoblast adhesion and viability after 24 h of culture in the TMT device inside and outside a CO_2_ incubator.** (A) Representative fluorescence images of myoblast adhesion throughout the whole scaffold on the PU scaffold in the TMT in and in the TMT out throughout the whole scaffold (top, middle, and bottom sections). Blue: nuclei (Hoechst) and PU scaffold. Scale bar = 100 μm. (B) Myoblast viability measured by LDH release normalized on the total DNA, reported in (C), and representing the number of cells on the scaffolds (Mann–Whitney test, ns = *p* > 0.05).

The sterilized PU scaffolds were coated with PDA (d-2) and FN (d-1); then, 1.5 × 10^6^ myoblasts were statically seeded on each scaffold (d0). The homogeneity of cell suspension dispersion was ensured by two seeding sessions, which were also done to counterbalance any possible gravity-related effect that would prevent a homogeneous scaffold seeding. After 5 h in a CO_2_ incubator, the TMT devices were connected to the peristaltic pump by means of the two tendon-like channels. GM was perfused from the bottom side to the top one for 24 h at 0.62 mL/min. Two conditions were explored during the experimental phase (d1): a set of devices was placed inside the CO_2_ incubator (TMT in), whereas another one was placed outside in air on the bench (TMT out). Figure 9A shows the presence of myoblasts, counterstained for their nuclei, attached throughout the whole PU scaffold in both conditions. No differences were observed between the two experimental conditions, with a high-density cell coverage of the PU trabeculae both in the TMT in and in the TMT out. Cell viability was assessed through the release of LDH in the culture media normalized on the total DNA amount of each sample (Figure 9B,C). The LDH release measurements demonstrated that there was no significant difference between the viability of myoblasts kept in the TMT *in* and those growing in the TMT out, kept in air. Consequently, these results attest that the TMT device is functioning properly in maintaining cell constructs alive in air, by keeping the cells in physiological conditions.

Altogether, these results demonstrate that the proposed device is suitable for keeping myoblasts alive in a 3D scaffold in culture outside a CO_2_ incubator for 24 h, for potential applications as a platform for biohybrid robots in air. Indeed, the results are comparable with the ones obtained for myoblasts growing on the same support but in a canonical environment for cell culture (37 °C, 5% CO_2_).

## 4 Conclusions

In this study, we developed a flexible perfusion device to address two major bottlenecks currently hampering the translation of biohybrid soft robots from laboratory research to real-world applications, namely, the transition from 2D to 3D systems and the development of life-sustaining systems to maintain the functionality of mammalian cells even if removed from the incubator.

The TMT device had a stiffness (*E* ∼5.9 kPa) and a protein composition (fibronectin coating) close to the natural muscle tissue. A compliant silicone membrane prevented medium evaporation, and two tendon-like hollow channels allowed nutrient supply. The device allowed for safely maintaining a 3D cell culture of C2C12 cells under constant perfusion and in air (*i.e.*, out of the CO_2_ incubator) for 24 h.

Future experiments will focus on evaluating the performance of the device for a more extended period and on integrating external stimuli (*e.g.*, mechanical stimulation) to further boost muscle tissue development.

## Supporting Information Available

Mesh convergence analysis (Figure S1); peristaltic pump calibration with the TMT (Figure S2); permeability measurements of the polyurethane scaffold (Figure S3); and wall thickness measurements of the PDMS cylindrical membrane (Figure S4) (PDF)
