# Supplementary material for: Soft Perfusable Device to Culture Skeletal Muscle 3D Constructs in Air
Source: ACS Appl Bio Mater. 2023 Jun 21;6(7):2712–24. doi: 10.1021/acsabm.3c00215 (PMC10354741; doi:10.1021/acsabm.3c00215)
Supplement: Supplementary file 1 — mt3c00215_si_001.pdf [file mt3c00215_si_001.pdf]

Supporting Information

Soft perfusable device to culture skeletal muscle 3D constructs in air

Federica Iberite [1,2,+], Marco Piazzoni [3,4,+,], Daniele Guarnera[1,2], Francesco Iacoponi[1,2], Silvia Locarno[3], Lorenzo Vannozzi[1,2], Giacomo Bolchi[3], Federica Boselli[3], Irini Gerges[5], Cristina Lenardi[3], Leonardo Ricotti[1,2]*

[1] The BioRobotics Institute, Scuola Superiore Sant'Anna, Piazza Martiri della Libertà 33, 56127 Pisa, Italy

[2] Department of Excellence in Robotics & AI, Scuola Superiore Sant'Anna, Piazza Martiri della Libertà 33, 56127 Pisa, Italy

[3] Department of Physics, Università degli Studi di Milano, Via Celoria 16, 20133, Milano, Italy

[4] Department of Biomedical, Surgical and Dental Sciences, Università degli Studi di Milano, 20100 Milano, Italy

[5] Tensive S.r.l, Via Timavo 34, Milan 20124, Italy

+ Both these authors contributed equally to this work.

*Corresponding author e-mail address: marco.piazzoni@unimi.it

Figure S1: Mesh convergence analysis.

Figure S2: Peristaltic pump calibration with the TMT.

Figure S3: permeability measurements of the polyurethane scaffold.

Figure S4: Wall thickness measurements of the PDMS cylindrical membrane.

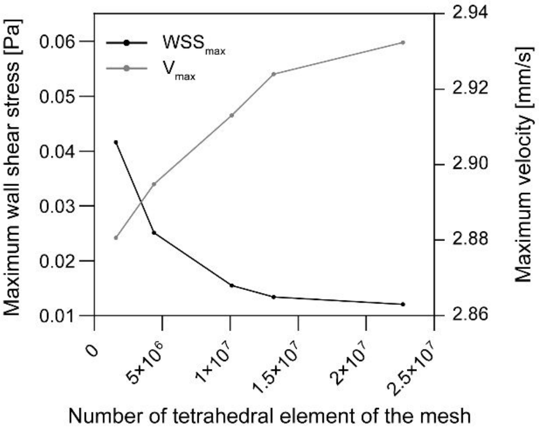

Figure S1: **Mesh convergence analysis**. Estimated values of maximum wall shear stress (WSS) (black line) and maximum velocity (gray line) by refining the mesh. The mesh size used for the computations was 10^{-5} m and the number of tetrahedral elements was ~ 1.1×10^7.

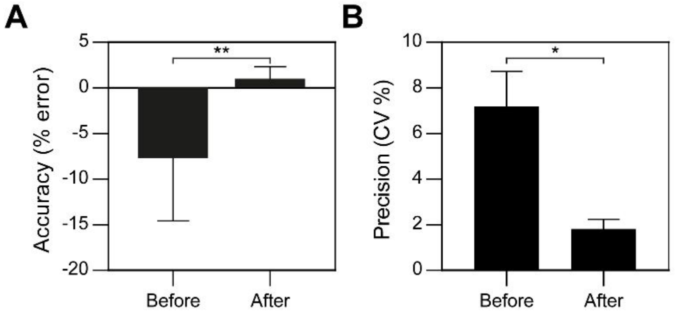

Figure S2: Peristaltic pump calibration with the TMT. A) Accuracy before and after the peristaltic pump calibration (Paired t-test, * $p \leq 0.05$). **B)** Precision of the actual flow rate, expressed as coefficient of variation (CV) before and after the peristaltic pump calibration (Paired t-test, ** $p \leq 0.01$). To evaluate the performance of the pump with the TMT bioreactors (N = 3), three independent measurements were carried out for each sample. The measurements were performed before and after the calibration process. In particular, the accuracy of the pump was determined by comparing the actual flow rate of the trial with the programmed flow rate:

$$\text{Accuracy} = \left(\frac{\text{Actual infusion rate } - \text{Programmed infusion rate}}{\text{Programmed infusion rate}}\right) \times 100\% \qquad \text{(S1)}$$

Pump precision was assessed as the coefficient of variation (CV) of the 3 trials performed.

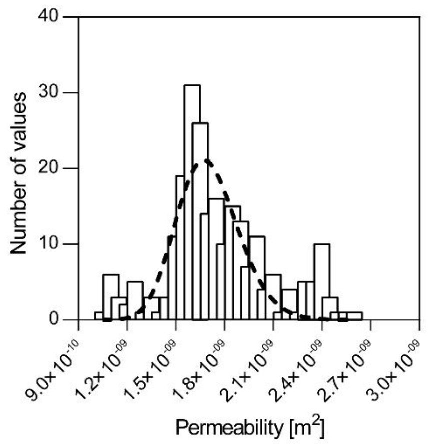

Figure S3: permeability measurements of the polyurethane scaffold. PU scaffold permeability (N = 3) was measured using the constant pressure gradient method [1] as described in Iberite *et al.* [2] The intrinsic permeability (k) was determined using Darcy's law (equation S2), where Q is the volumetric flow rate of water (estimated water density ρ_{water} = 998 kg/m^3 and viscosity η = 8.9×10^{-4} Pa s), L is the sample height, A is the sectional area of the tube, and $\Delta P = \rho_{water} H g$ (with H = height of the water above the sample):

$$\text{k} = \eta \frac{Q}{A} \frac{L}{\Delta P} \qquad \text{(S2)}$$

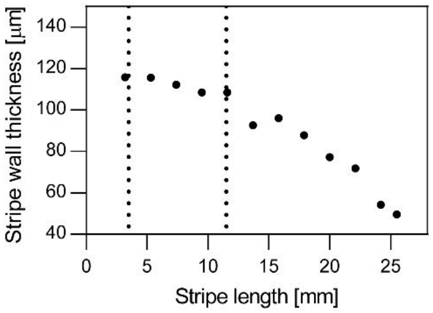

Figure S4: **Wall thickness measurements of the PDMS cylindrical membrane**. Representative wall thickness profile of a stripe cut out of the PDMS cylindrical membrane. Dashed lines indicate the portion of the PDMS cylindrical membranes that have been used to encapsulate the PU scaffold. A stripe (l = 25 mm; w = 2 mm) was cut out from the cylindrical membrane with a scalpel and laid on the edge of a microscope slide. The slide was positioned over the objective tilted by 90° to allow a side view of the stripe. Wall thickness was measured starting from the thin portion of the stripe every 2 mm using an image capturing software (ISCapture).

Supplementary References

[1] Varley M.C.; Neelakantan S.; Clyne T.W.; Dean J.; Brooks R.A.; Markaki A.E. Cell structure, stiffness and permeability of freeze-dried collagen scaffolds in dry and hydrated states. *Acta Biomater.* **2016**, 33, 166–175, doi: 10.1016/j.actbio.2016.01.041.

[2] Iberite F.; Gerges I.; Vannozzi L.; Marino A.; Piazzoni M.; Santaniello T.; Lenardi C.; Ricotti L. Combined Effects of Electrical Stimulation and Protein Coatings on Myotube

Formation in a Soft Porous Scaffold. *Ann. Biomed. Eng.*, **2019**, 48, 734–746, 2020, doi: 10.1007/s10439-019-02397-9.
